# Dengue Virus Surveillance for Early Warning, Singapore

**DOI:** 10.3201/eid1605.091006

**Published:** 2010-05

**Authors:** Kim-Sung Lee, Yee-Ling Lai, Sharon Lo, Timothy Barkham, Pauline Aw, Peng-Lim Ooi, Ji-Choong Tai, Martin Hibberd, Patrik Johansson, Seow-Poh Khoo, Lee-Ching Ng

**Affiliations:** Environmental Health Institute, Singapore (K.-S. Lee, Y.-L. Lai, S. Lo, L.-C. Ng); Tan Tock Seng Hospital, Singapore (T. Barkham); Genome Institute of Singapore, Singapore (P. Aw, M. Hibberd); Ministry of Health, Singapore (P.-L. Ooi); National Environment Agency, Singapore (J.-C. Tai, S.-P. Khoo); DSO National Laboratories, Singapore (P. Johansson)

**Keywords:** Dengue, surveillance, PCR, sequencing, phylogenetic, viruses, dispatch

## Abstract

In Singapore, after a major outbreak of dengue in 2005, another outbreak occurred in 2007. Laboratory-based surveillance detected a switch from dengue virus serotype 1 (DENV-1) to DENV-2. Phylogenetic analysis showed a clade replacement within DENV-2 cosmopolitan genotype, which accompanied the predominant serotype switch, and cocirculation of multiple genotypes of DENV-3.

Dengue poses a threat to public health in >100 countries worldwide. Despite improvements in diagnostics and clinical management, the number of dengue cases continues to rise globally; 2.5 billion persons are at risk for infection (*1*). Dengue virus (DENV) belongs to the genus *Flavivirus* and contains a positive-strand RNA genome that encodes 3 structural proteins—core protein, membrane-associated protein, envelope protein—and 7 nonstructural proteins. DENV consists of 4 genetically and antigenically distinct serotypes, 1–4.

Singapore has seen a resurgence of dengue cases since the late 1980s, after 2 decades of successful control that relied mostly on an integrated vector-control program (*2*,*3*). The recent epidemiology of dengue in Singapore is characterized by a 5–6-year cycle; incidence rates increase within each cycle before collapsing into 1 or 2 lull years. During an unprecedented dengue outbreak in 2005, a total of 14,006 cases and 27 deaths were reported (*4*). The outbreak was associated with a switch in predominant serotype, from DENV-2 to DENV-1, in 2004 (*5**,**6*). In 2007, Singapore experienced another dengue outbreak after a lull in 2006. We report the laboratory and surveillance findings that assisted vector-control operations during the 2007 dengue outbreak in Singapore.

## The Study

In Singapore in 2005, as part of an integrated vector-control program, laboratory-based dengue virus surveillance was established for close monitoring and investigation of the circulating dengue virus serotypes. Samples were sent to the Environmental Health Institute from Tan Tock Seng Hospital, which cares for ≈40% of all reported dengue patients in Singapore, and from a network of participating general practitioners throughout the country. PCR to detect dengue virus RNA and serotyping were performed at the Environmental Health Institute according to its in-house real-time PCR protocol (*7*). The numbers of dengue-positive samples serotyped were 186 in 2006, 889 in 2007, and 918 in 2008, and represent ≈10% of the total dengue cases reported each year by the Ministry of Health.

The envelope protein gene of DENV (≈1,480 nt) was amplified by reverse transcription–PCR and directly sequenced by using an automated DNA sequencer (ABI 3100; Applied Biosystems, Foster City, CA, USA). Sequences were aligned and submitted to GenBank (accession nos. GQ357666–892). Phylogenetic analysis of DENV sequences was conducted by using the maximum-likelihood method as implemented in PAUP* software, version 4.0b10 (*8*), and compared with sequence data obtained from GenBank.

**Figure 1:**
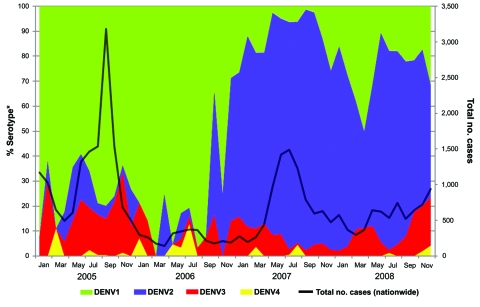
Trends of monthly dengue cases in Singapore, 2005–2008, showing a switch in predominant serotype from dengue virus serotype 1 (DENV-1) to DENV-2 in January 2007 and cocirculation of all 4 serotypes with general dominance of DENV-1 and DENV-2 and lesser circulation of DENV-3 and DENV-4. *From ≈10% of all dengue cases.

During 2006–2008, all 4 DENV serotypes were detected (Figure 1). DENV-1 (21.7%) and DENV-2 (69.3%) were the predominant serotypes throughout the study period; DENV-3 (7.8%) and DENV-4 (1.2%) were less prevalent. In 2006, the number of DENV cases was relatively low, and DENV-1 remained the predominant serotype after the major 2004–2005 outbreak. During January–September 2006, 75%–100% of samples collected each month contained DENV-1. In early January 2007, the predominant circulating serotype switched from DENV-1 to DENV-2. Early detection of this switch warned of a possible upcoming dengue outbreak. In response, an enhanced vector-control program was activated in February 2007. The proportion of DENV-2–positive samples detected by PCR rose from 57.9% in January 2007 to a peak of 91.0% in July 2007. This increase was accompanied by an increase in the total number of dengue cases reported by the Ministry of Health; cases peaked at 432 in the first week of July 2007. By late August, the number of dengue cases fell to below the warning level (warning level = 256 cases/epidemiologic week) as reported by the Ministry of Health (*9*). During the switch in predominant serotype, fatality rates (0.32% in 2006 and 0.27% in 2007) and dengue hemorrhagic fever rates (2.4% in 2006 and 2.1% in 2007) did not differ substantially among the reported cases. During this same period of extensive surveillance, 5.2% of the samples in 2007 and 10.8% in 2008 were detected as DENV-3. Our spatial analysis indicated localized emergence of DENV-3 in the eastern region of the country in 2007 and in the central region in 2008. Enhanced control was also attempted in these areas to prevent the spread of the serotype that had been uncommon in Singapore.

**Figure 2:**
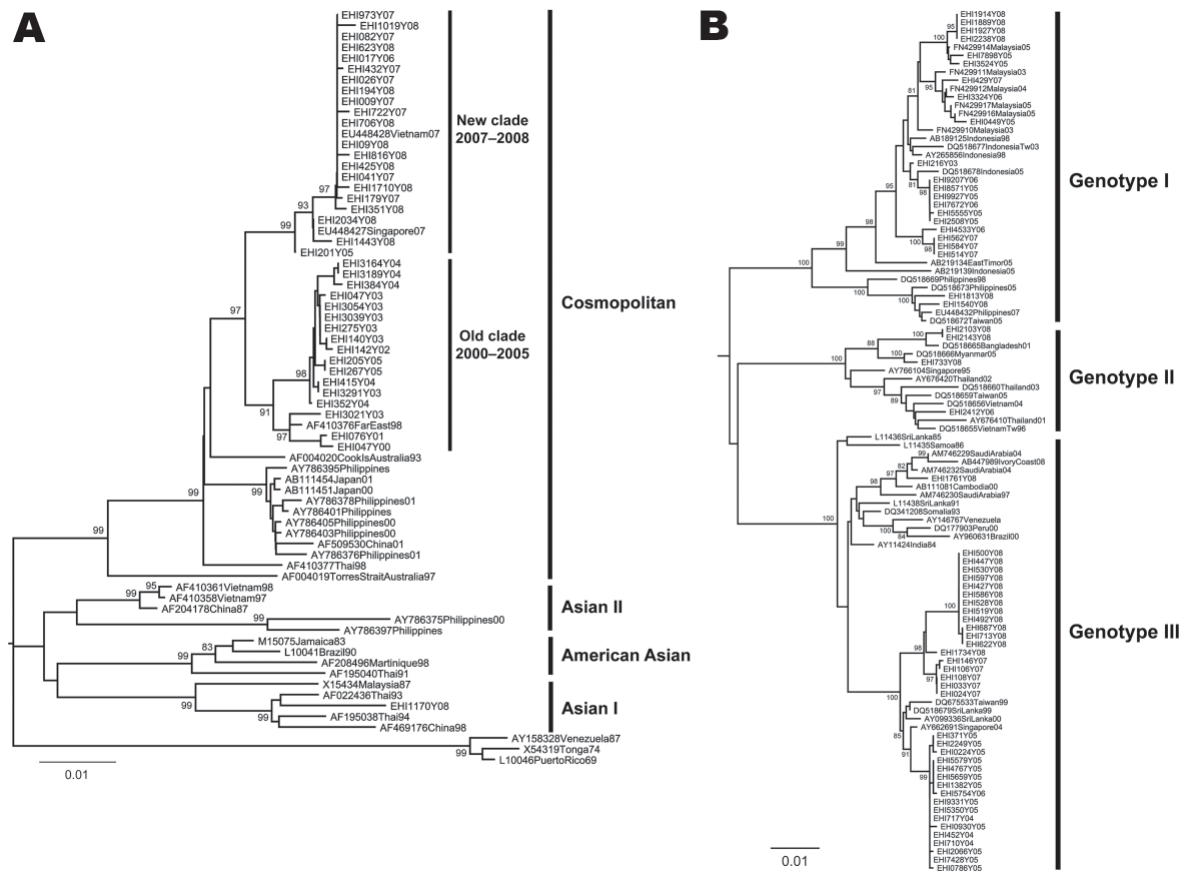
Maximum-likelihood tree showing the phylogenetic relationship of A) dengue virus serotype 2 (DENV-2) and B) DENV-3 from Singapore and global isolates based on the envelope protein gene. EHI, sequence data generated at Environmental Health Institute; new clade, isolates obtained in 2007 and later; old clade, isolates obtained before 2007. Numbers on branches represent bootstrap percentages; only those >80% are shown. Scale bars indicate substitutions per site.

Phylogenetic analysis of DENV-2 envelope gene sequences showed that the switch in predominant serotype in early 2007 coincided with a clade replacement within DENV-2. During 2000–2008, 2 distinct subclades, with strong temporal topology, were found within the cosmopolitan genotype (Figure 2). Specifically, DENV-2 isolates obtained before 2007 formed the subclade herein referred to as the old clade, whereas isolates obtained in 2007 and later formed the new clade with strong bootstrap support. Because 1 of the DENV-2 isolates sampled in 2005 clustered with the new clade but fell closer to the root of that clade, in situ evolution giving rise to DENV-2 viruses that subsequently replaced the old clade viruses is highly likely. A GenBank sequence that belonged to DENV-2, sampled in 2007 in Vietnam, grouped within the new clade, indicating that this virus strain was not restricted to Singapore and may have been circulating in this region.

Our dengue surveillance also indicated sporadic emergence of DENV-3 from localized areas throughout the country (*6**,**10*). Phylogenetic analysis of isolates from Singapore from 2006 through 2008 identified 3 genotypes of DENV-3. These isolates were closely related to those found in Indonesia, Malaysia, Philippines, Thailand, Saudi Arabia, and Côte d’Ivoire (Figure 2), which suggests multiple importations of DENV-3 viruses into Singapore. Analysis of DENV-1 sequences showed that all except 3 belonged to genotype I and were similar to those responsible for the 2005 outbreak (data not shown).

## Conclusions

Our dengue surveillance provided early warning of the outbreak in 2007 and contributed to early activation of enhanced vector control. Although we were unable to assess the effectiveness of the control measures, considering the regional situation in 2007 (*11**,**12*), we believe that without these measures the dengue situation in Singapore in 2007 would have been worse than or comparable to that in 2004–2005. After a lull year in 2006, dengue cases were expected to rise for a few years. The integrated vector control program has interrupted the dengue trend, with 7,032 cases reported in 2008 and 4,498 in 2009.

As a travel hub, Singapore experiences continuous importation of dengue viruses. Although some become established at various levels, some develop into outbreaks and subsequently get replaced. Our study demonstrates how rapidly dengue virus serotypes can be replaced within a population. It also highlights the complexity of the disease and the challenges faced by affected states that seek to understand the epidemiology for purposes of disease control. To shed further light on the complex interplay among the various factors that affect dengue transmission, studies are being conducted on complete genome sequences of dengue viruses, vectorial capacity of local *Aedes* spp. mosquitoes, and cross-reactive immune responses to different dengue serotypes.
